# The Box Interaction Game: Action-Based Divergent Thinking Tests for Chinese Preschoolers

**DOI:** 10.3390/jintelligence13070075

**Published:** 2025-06-24

**Authors:** Ying Du, Yiduo Xiao, Haoran Yang, Yunqi Ning, Fei Zhi, Jing Chen, Yinghui Guo, Qunlin Chen

**Affiliations:** 1 Faculty of Psychology, Southwest University, Chongqing 400715, China; 2 School of Psychology, Hainan Normal University, Haikou 571158, China; 3 School of Public Policy and Administration, Chongqing University, Chongqing 400044, China; 4 Center for Studies of Education and Psychology of Ethnic Minorities in Southwest China, Southwest University, Chongqing 400715, China

**Keywords:** preschoolers, creative potential, divergent thinking, exploratory games

## Abstract

The current methodologies for assessing divergent thinking in children are predominantly based on verbal response, which limits their applicability for evaluating the creative potential of preschoolers and toddlers. This study introduces the Box Interaction Game (BIG), which is an adaptation of the Unusual Box Test (UBT) to make it more suitable for Chinese children. By simplifying, reorganizing, and expanding the actions in the UBT, the BIG employs action-based assessments that are relevant to the Chinese context and evaluate validity and test-retest reliability in preschoolers. The results revealed statistically significant but modest correlations between the verbal Unusual Uses Task (UUT) and the BIG test. Specifically, total scores (*τ* = 0.24, *p* = 0.02), fluency scores (*τ* = 0.23, *p* = 0.029), and originality scores (*τ* = 0.21, *p* = 0.04) showed low-to-moderate associations, indicating preliminary support for convergent validity, although further refinement is needed to strengthen these relationships. Additionally, the BIG demonstrates strong internal consistency (Cronbach’s alpha = 0.83 for both fluency and originality) and moderate test-retest reliability (ICC for fluency = 0.67, for originality = 0.74). These findings suggest that BIG is a promising and developmentally appropriate tool for assessing divergent thinking in Chinese preschoolers, offering a foundation for future work on early creative thinking in China.

## 1. Introduction

Creativity, a fundamental cognitive trait crucial for human adaptation, plays a pivotal role in learning, career development, and daily life. It is commonly defined as the ability to generate novel, unique, and valuable ideas or products in response to specific stimuli (Runco and Jaeger 2012; Sternberg and Lubart 1999). Some perspectives view creativity as a holistic construct, integrating personality, process, product, and environment (Batey 2012). Divergent thinking has long been considered a key component of creativity and is the most commonly used indicator of an individual’s creative potential. Divergent thinking, which involves generating many possible solutions to a problem, fosters diverse and novel ideas, potentially leading to creative problem-solving approaches (Barbot et al. 2016). Despite extensive research on divergent thinking development, most studies focused mainly on school-age children, adolescents, and adults by using verbal and figural creative thinking measurements (Bai et al. 2023). Few studies have explored divergent thinking in early childhood (e.g., preschoolers) primarily due to a lack of valid and user-friendly tools (Hoicka et al. 2013). This study aims to improve the Unusual Box Test (UBT) for assessing divergent thinking tailored to Chinese preschoolers. By examining creative thinking in preschoolers, we can better understand its emergence and lay the groundwork for nurturing creativity and innovation in children.

In the field of creativity, divergent thinking tests can be broadly categorized into verbal tests, figural tests, and action-based assessments, which evaluate performance through physical movements, such as those involving the hands and feet. However, some tests often prove unsuitable for preschoolers (Bijvoet-van Den Berg and Hoicka 2014). Most require verbal responses, which are impractical for young children who cannot express their thoughts fluently and clearly, let alone write them down. Additionally, abstract concepts such as “novel”, “be creative”, and “unusual”, central to many creativity tests, are difficult for young children to grasp (Bai et al. 2021; Hoicka et al. 2013). This may pose a cognitive challenge for children, potentially reducing the number of responses and impacting the test’s effectiveness. Moreover, in many action-based divergent thinking tests, young children tend to imitate rather than create new actions, influenced by examples provided or previously learned behaviors (Flynn 2008). As children’s age decreases, the accuracy of current testing methods does not yield good results. Numerous studies have identified challenges in applying traditional divergent thinking assessments, such as the Torrance Tests of Creative Thinking (TTCT) and the Unusual Uses Task (UUT), to younger children, as these tools were originally developed for older children and adults. Kaufman and Baer (2005) note that traditional creativity assessment tools may lack the sensitivity to accurately capture the creative abilities of very young children(Kaufman and Baer 2005). For young children, there is a need to develop specialized assessment instruments that better align with their cognitive and linguistic developmental stages. Additionally, researchers must consider variations in language proficiency, comprehension, and expression styles to ensure the accuracy and reliability of assessment outcomes. In summary, existing measurements may be insufficient for effectively assessing the creativity of children under six, making it challenging to explore the developmental trajectories of creativity in early childhood.

To address these limitations, Hoicka et al. (2013) developed the UBT, an assessment of divergent thinking based on children’s actions during play. The UBT presents children with a multifaceted box and several unfamiliar objects, encouraging play without relying on verbalization or predefined problem-solving tasks. Raters assessed the children’s creativity by carefully analyzing the movements and interactions captured in videos of the children engaging with the box. The UBT showed good test-retest reliability with preschoolers and toddlers (Bijvoet-van Den Berg and Hoicka 2014; Hoicka et al. 2013). This reliability is primarily due to the fact that the test observes actions as play, aligning with the natural exploratory behaviors of young children. Several empirical studies have validated the UBT as an effective tool for assessing divergent thinking in preschoolers (Hoicka et al. 2018; Wang et al. 2021). It also demonstrated significant correlations with other divergent thinking tasks, such as the Instances Task and the Thinking Creatively in Action and Movement test (Bijvoet-van Den Berg and Hoicka 2014). Piaget indicated that exploration naturally occurs during play (Piaget 2017). Previous research has shown that children who actively explore objects during play perform better on divergent thinking tasks compared to those who passively observe (Lloyd and Howe 2003). Consequently, the diversity and quantity of actions young children exhibit while playing can serve as indicators of divergent thinking.

In the research of UBT, the tool was originally designed and tested for children in Western societies. According to Csikszentmihalyi’s systems model of creativity, creativity arises from the dynamic interaction among individuals, cultures, and environments (Csikszentmihalyi 2015). It is crucial to understand and evaluate creative processes and actions within different culture contexts (Bijvoet-van Den Berg and Hoicka 2014; Chen et al. 2023; Fogarty et al. 2015). Cross-cultural studies have shown differences in creativity between individuals in Eastern and Western cultures, particularly in divergent thinking, where Westerners tend to exhibit more significant advantages (Guo et al. 2021; Morris and Leung 2010). The UBT, designed based on the psychological development characteristics of Western children, may not be entirely suitable for Chinese children due to cultural differences. We developed a set of tools based on the UBT and conducted preliminary experiments involving six preschoolers. We observed that some actions recognized by the original UBT scoring system were rarely seen in Chinese children, while new actions unique to the Chinese context emerged. Additionally, children lacked interaction with the UBT box and its items. Based on these observations, we recognized the necessity of adapting the UBT and developing a new scoring system tailored to our specific context. This adaptation aims to ensure that the test is more suitable for our cultural background and the cognitive characteristics of our children, while also enhancing the consistency and accuracy of the scoring. Therefore, making adjustments and local improvements to the UBT is essential. These modifications will ensure the test’s validity and applicability for Chinese preschoolers and provide a valuable research approach to explore potential cultural differences in children’s creative thinking.

Furthermore, the interpretation of actions and the scoring scheme in Chinese contexts are not well aligned with those of Western cultures. Translating the scoring scheme presents significant challenges. For example, in English, the action “pull” is understood as pulling part of the box or object toward the participant. In Chinese, “拉 (LA)” can mean pulling a part of the box towards the participant, pulling both ends of an object in different directions, or overlapping with synonyms like “扯 (CHE),” which need to be distinguished. This difference mainly stems from the characteristics of the English and Chinese languages. English verbs often have multiple meanings, with some phrasal verbs having dozens of interpretations (Kramsch 2014). In contrast, Chinese, an isolating language with logographic characters, lacks inflections and uses a flexible word order to express various concepts (Palmer and Wu 1995). Some scholars note that “Chinese phrasal verbs are almost always monosemous” due to their ad-hoc combinatory nature (Kramsch 2014). In the UBT scoring scheme, an action word in English may correspond to multiple Chinese words, with associated actions not reflected in the original. Additionally, Chinese children exhibited actions that were not accounted for in the original scoring system. For example, during the pre-experiments, children frequently exhibited “CHUO” (戳) behavior—defined as forcefully thrusting the tip of an elongated object to penetrate or make contact with hollow materials— which were not recognized by the UBT’s original scoring system. Conversely, some actions that are common in the UBT’s scoring system are rarely observed among Chinese children. Research focusing on adults, divergent thinking, and cultural differences suggests that Western individuals may perform better in divergent thinking tasks, highlighting the impact of cultural differences on creativity. The variations in children’s behavior observed in our pre-experiments reflect the unique cultural context, indicating that the original scoring criteria are not fully applicable to our environment. To apply the UBT to Chinese children, it is necessary to redefine action categories, expand the scoring sheet coverage of actions, and add more nuanced scoring rules that account for the richness of Chinese vocabulary.

Based on the UBT, this study aims to develop a Box Interaction Game (BIG) test tailored for Chinese preschoolers. We revised the scoring scheme for interactive actions during the game, adding and refining action categories to better align with the behavioral habits of local children. To examine the validity and test-retest reliability of the BIG test for preschoolers, we conducted tests on children aged 3 to 6, simultaneously measuring two divergent thinking tests assessed by verbal responses: the Unusual Uses Test (UUT) and the Pattern Meanings Test (PMT). We administered the BIG test to the same group of children 1 month later to evaluate its test-retest reliability. We expected the BIG test to capture the divergent thinking abilities in preschoolers and to demonstrate that this ability is stable over time. Additionally, we investigated gender differences in divergent thinking and the relationship with age among preschoolers.

## 2. Method

### 2.1. Participants

A total of 47 preschoolers (24 females, aged 3 to 6.5 years, M = 4.84, SD = 1.05) were enlisted from a kindergarten in Chongqing. Twenty of the participants (twelve females) took part in the retest, which occurred 1 month after the initial experiment. The research was ethically endorsed by the ethics committee of Faculty of Psychology at Southwest University (reference number: H23109). The kindergarten administration willingly approved the project and provided the necessary space for the experiments. Prior to the study, the parents or guardians of each child signed consent forms. As a token of appreciation, the young participants received toys or books valued at CNY 50 to 60.

### 2.2. Materials

#### 2.2.1. The BIG Box

**Figure 1:**
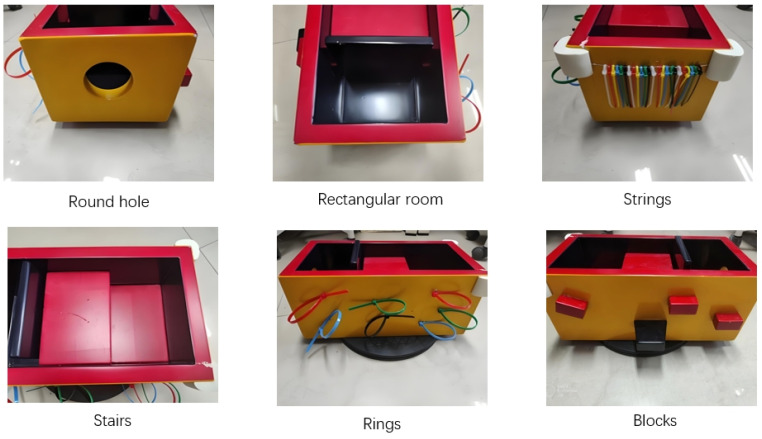
The wooden box.

**Figure 2:**
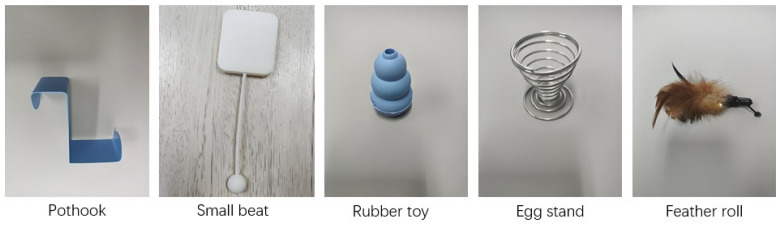
The five novelty toys.

Based on UBT, the BIG box is a wooden box featuring ledges, ropes, rings, stairs, black barriers, and round holes (see Figure 1). The revision mainly focuses on improving safety and functionality. First, in terms of safety, plastic sponges have been installed on the sharp edges of the box to prevent accidental injury. In addition, one of the five novelty items, the potentially dangerous mixing spoon, has been replaced by a more child-friendly paddle-shaped tool with a ball on the end, significantly reducing risk during the testing phase (see Figure 2). Second, to improve accessibility, the BIG box is mounted on a rotating base with a diameter of 25 cm. This modification allows children to easily access and explore all aspects of the device from any angle, encouraging interactive play and learning. Finally, based on data from informal testing, it was observed that children frequently moved in areas that had not received attention. To address this, we adjusted the device’s structure by reducing the width of the black barrier on one side, creating a “black barrier gap” and clearly marking the black barrier and iron wire as key observation points for more comprehensive behavioral analysis. These carefully designed changes aim to provide a safe and educational environment that promotes children’s cognitive development exploratory behavior.

#### 2.2.2. Pattern Meanings Test

The Pattern Meanings Test (PMT), adapted by Elena Hoicka from Wallach and Kogan’s tests of creativity (Wallach and Kogan 1965) evaluates children’s cognitive flexibility and creative problem-solving abilities. Although there are few studies directly mentioning the name “PMT,” similar graphic interpretation tasks have been used in Chinese research. In this test, children view four line drawings and provide as many interpretations as possible, describing what each drawing “is” or “reminds them of.” The test is completed within 10 min, with prompts given if responses cease. Scoring is based on three dimensions: (i) fluency, which is the number of responses for an item after excluding those ideas that were inappropriate and difficult to understand; (ii) flexibility, which is evaluated by counting the number of different categories of responses,; a category refers to a distinct interpretation or classification of the line drawings provided, for instance, if a child interprets a drawing as both an animal and a vehicle, these would count as two different categories; and (iii) originality, which is assessed based on the frequency distribution of responses in the whole sample. All responses are gathered and the frequency of each response is tallied. Scores are assigned as follows: five points for responses occurring within the 0–5% range, four points for responses within the 5–20% range, three points for responses within the 20–50% range, two points for responses within the 50–70% range, and one point for responses appearing more frequently than 70% (Jung et al. 2010). To ensure comparability across measures, all scores (originality, flexibility, fluency) were converted to z-scores for scale compatibility and the total creativity score was calculated as their sum.

#### 2.2.3. Unusual Uses Task

The Unusual Uses Task (UUT) was employed to assess individual divergent thinking in this study. The use of UUT in China is more widespread, with many studies employing this tool (Sternberg 2003; Guo et al. 2021; Runco and Acar 2012). In this task, children were asked to generate as many interesting and uncommon uses as possible for two common objects (i.e., a can and a cardboard box). At the beginning of the experiment, an experimental assistant provided the following instruction: “I will show you two familiar objects, and you need to think of as many different uses for these items as possible.” The experimenter then demonstrated an example using a newspaper, suggesting some unconventional uses, such as folding it into a hat. Once the children understood the task and generated a new idea, the formal UUT test was administered. During the test, the experimenter encouraged the children to come up with additional answers (e.g., “Can you tell me more about this object?”) until they could no longer think of any, at which point they moved on to the next item. Throughout the experiment, an experimenter recorded the participants’ responses in real-time and supplemented this with a recording pen. The entire process was completed within 5 min. The test utilized the same scoring dimensions and methods as the Pattern Meanings Test (PMT).

### 2.3. Procedure

**Figure 3:**
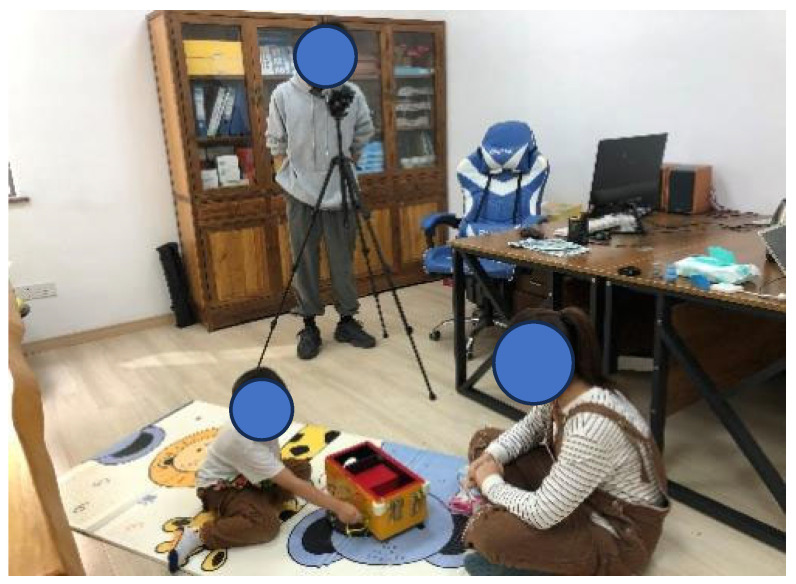
An example of a field test.

Two experimenters were trained to administer these tests, which were all conducted in a separate room within the kindergarten (see Figure 3). All children were required to complete three tests: BIG, UUT, and PMT. Since UUT and PMT are verbal response tasks, they were considered as a single unit and the order of administering BIG and the verbal tasks was balanced. Each child was brought to the room by their teacher and introduced to the two experimenters. The teacher would say something like, “These two big brothers or sisters will play with you for a while. They have some fun games.” During the administration of the tests, one of the experimenters was responsible for reading the instructions and ensuring the child understood the tasks by asking simple questions. The other experimenter was responsible for video and audio recordings.

#### Procedure for the Box Interaction Game

The experimenters placed the BIG box on a turntable to ensure each side was easily accessible to the children. The entire setup was positioned in the center of a floor mat to provide a designated play area. One experimenter explained the task to the children, informing them that they would be playing a game with the box while remaining on the edge of the mat. The children were encouraged to freely explore the box and its features. If a child did not actively engage with the box within a certain time, the experimenter would gently rotate the box, exposing each feature for no more than 10 s to encourage further interaction. After this rotation, the child was allowed to continue playing with the box and another toy until instructed otherwise. Each child was given one of five novelty items to interact with, allowing 120 s for exploration before they were introduced to a new object.

### 2.4. Coding

**Figure 4:**
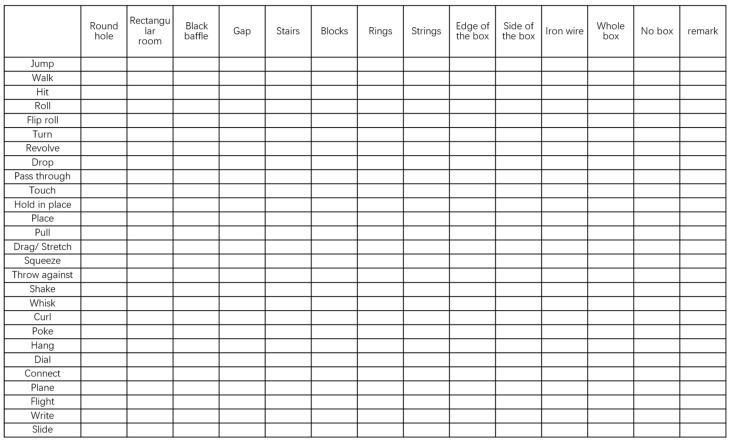
Scoring table including actions and action locations.

These interactions with the BIG box and novel objects were coded to evaluate divergent thinking ability in terms of originality and fluency. Based on the performance of the children in the pre-experiment, we identified a set of movements commonly exhibited by Chinese children. We compared these movements with the scoring criteria of the UBT test and ultimately identified 23 movements. Of these, 13 were retained from the UBT, while 14 new movements were added, which better align with the definitions and observability within the Chinese culture context. Figure 4 shows the scoring matrix for the BIG test, comprising 27 actions and 13 positions on the box (e.g., stairs, round hole). The new action is marked in yellow and the new position is marked in green. Each cell in the matrix represents the potential actions a child might perform with an object in different positions. For example, if a child uses a novel toy to tap on the stairs of the box, “tapping” is the action and “stairs” is the position (see Appendix A). Each novel toy corresponds to one scoring matrix, and each child has five scoring matrices as their results, one for each of the five novel toys used in the experiment. Additionally, the use of novelty objects without the box was also counted, marked as “out of the box” within the box. For repeated actions, points are only awarded if the action, position, or object changes; repeating the same action in the same position does not score. During scoring, raters thoroughly watched each child’s video for each object, logging actions on the scoring matrix. Each rater learned the definition of each action (see Appendix B).

Based on all children’s actions across the five objects, fluency and originality were calculated for each child. Fluency refers to the number of different cells filled in the matrix for each object. Originality was evaluated by calculating the occurrence probability for each action within each position (cell). Actions with an occurrence probability of less than 5% were scored three points, those between 5% and 20% were scored two points, and all others were scored 1 point. For each child playing with each object, originality is the sum of the originality scores for each cell and total originality is the sum across all five objects. To ensure comparability across measures, all scores (originality and fluency) were converted to z-scores for scale compatibility and the total creativity score was calculated as their sum. Three psychology majors conducted the coding and scoring. Before formal scoring, they underwent training to proficiently discern children’s movements, differentiate easily conflated actions, and identify composite movements, as well as pre-scoring practice. The scoring process was performed under the supervision of the first author.

## 3. Results

### 3.1. Descriptive Statistics

Table 1 displays the descriptive statistics for the dimensions scores of each test. We categorized 47 preschoolers into three age groups: 3–4 years old, which included 14 children; 4–5 years old, comprising 11 children; and 5–6.5 years old, with 22 children included.

### 3.2. Inter-Rater Reliability and Test-Retest Reliability of the BIG Test

In the pre-test (N = 47), three raters participated in scoring fluency and originality for each object. The inter-rater reliability (Cronbach’s alpha) for fluency and originality scoring was 0.957 and 0.945, respectively. In the post-test (N = 20), the inter-rater reliability (Cronbach’s alpha) for fluency and originality scoring was 0.968 and 0.959, respectively. Internal consistency analysis of the five novelty items revealed equally robust reliability, with Cronbach’s alpha = 0.832 (95% CI [0.738, 0.896]) for fluency and Cronbach’s alpha = 0.832 (95% CI [0.739, 0.897]) for originality. Test-retest reliability was analyzed in fluency and originality across time 1 and time 2. For fluency, the ICC = 0.67, 95% CI [0.15, 0.87], *p* = 0.004. For originality, the ICC = 0.74, 95% CI [0.33, 0.90], *p* = 0.003, suggesting moderate reliability for the BIG test in children aged 3–6 years. These findings indicate that the BIG test demonstrates moderate to high inter-rater reliability, internal consistency, and test-retest reliability in assessing fluency and originality among 3–6-year-old children, supporting its effectiveness as a reliable assessment tool.

### 3.3. Split-Half Reliability for BIG Test

The BIG test retains the original 13 action definitions from the UBT and adds 14 actions that Chinese children perform frequently, totaling 27 actions. We aimed to examine the relationship between the existing 13 actions and the newly added 14 actions, as well as their contributions to the total scores in terms of fluency and originality. Therefore, we divided the results into two halves for the correlation test. The results showed that the fluency scores of the old actions were strongly correlated with the fluency scores (*r* = 0.53, *p* < 0.001) and the originality scores (*r* = 0.54, *p* < 0.001) of the new actions. Additionally, the originality scores of the old actions were moderately correlated with the fluency scores (*r* = 0.45, *p* = 0.002) and marginally correlated with the originality scores (*r* = 0.28, *p* = 0.057) of the new actions.

### 3.4. Validity of the BIG Test

**Figure 5:**
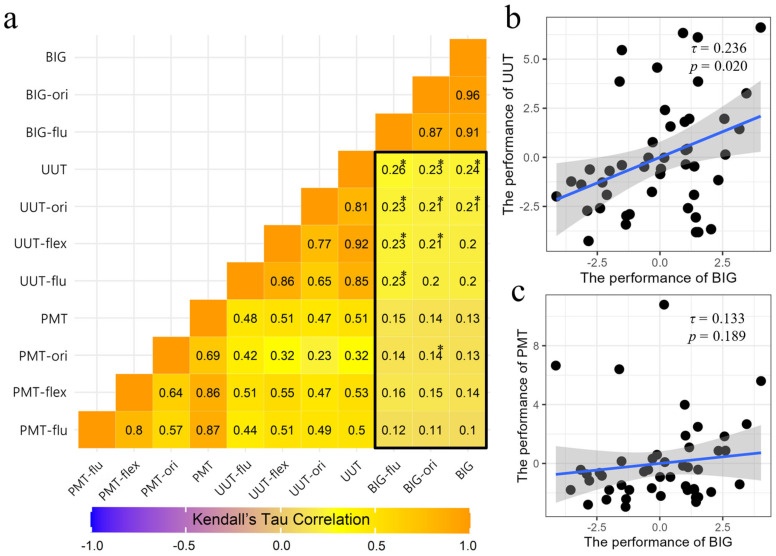
Correlation analysis of dimensions and total scores of the BIG test, AUT, and PMT: (**a**) Kendall’s Tau Correlation Heatmap; (**b**) Performance Correlation Between UUT and BIG; (**c**) Performance Correlation Between PMT and BIG Note: PMT, Pattern Meanings Test; UUT, Unusual Uses Task; BIG, Box Interaction Game; flu, fluency; flex, flexibility; ori, originality. * *p* < 0.05.

As shown in Figure 5a, the results of the Kendall rank correlation analysis indicate significant associations between the BIG test and the UUT scores across different dimensions. Specifically, BIG fluency was significantly correlated with UUT fluency (*τ* = 0.23, *p* = 0.029) and BIG originality was significantly correlated with UUT originality (*τ* = 0.21, *p* = 0.04). Furthermore, the total score of the BIG test was significantly correlated with the total score of the UUT (*τ* = 0.24, *p* = 0.02; see Figure 5b). While these findings provide initial support for the convergent validity of the BIG test, the relatively low correlation coefficients suggest that further refinement is needed to enhance its alignment with established measures. Additionally, the BIG test showed only weak and non-significant correlations with the fluency and flexibility scores of the PMT, as well as with the PMT total scores (*τ* = 0.133, *p* = 0.189; see Figure 5c), indicating limited overlap with this particular task.

Furthermore, we examined the relationships between the total scores of the old and new actions in the BIG test and the total scores of the UUT and PMT. The results showed that the total score of the old actions was not significantly correlated with either the UUT (*r* = 0.229, *p* = 0.121) or the PMT (*r* = −0.059, *p* = 0.694). In contrast, the total score of the new actions demonstrated a significant positive correlation with the UUT (*r* = 0.418, *p* = 0.003) and a moderate correlation with the PMT (*r* = 0.295, *p* = 0.044). These findings suggest that the BIG test possesses a certain degree of criterion-related validity. The significant correlation between the BIG test’s total score and other established creativity measures (such as the UUT) further supports its validity and highlights its potential as an effective assessment tool.

## 4. Discussion

The UBT has demonstrated its utility in measuring divergent thinking for preschoolers and toddlers, with its primary advantage being the capability to evaluate their divergent thinking ability through observation of their actions in non-verbal, action-based contexts, rather than through speech. The BIG test, developed as an adaptation of the UBT, has been modified to better suit the assessment of divergent thinking in Chinese preschoolers by reorganizing existing actions within the scoring sheet and adding new actions. The study confirmed the BIG test’s reliability, with moderate test-retest reliability and satisfactory inter-rater reliability. Importantly, scores calculated from the existing actions were strongly correlated with the scores of the new actions and both contributed equally to the overall divergent thinking score, suggesting that the inclusion of culturally appropriate actions as new items is suitable for evaluating divergent thinking in Chinese preschoolers. Furthermore, our results show that the BIG effectively captures divergent thinking, as evidenced by statistically significant—although modest—correlations between its fluency and originality scores and those on the UUT. These findings provide preliminary validation for its use in assessing creative thinking in early childhood.

Given the small sample size, the BIG test demonstrated moderate test-retest reliability and split-half reliability. These levels of reliability indicate that the BIG test can consistently measure divergent thinking abilities, although the modest reliability coefficients suggest that further refinement may be necessary. The alignment between the BIG and UBT tests supports the potential for the BIG test to assess divergent thinking effectively, but future studies with larger samples are needed to confirm these findings. Despite the moderate reliability observed, the consistency between the BIG and UBT tests suggests that the BIG test aligns well with young children’s natural exploratory behaviors during play (Bijvoet-van Den Berg and Hoicka 2014; Hoicka et al. 2013). However, due to the small sample size, further research is required to fully establish the stability and validity of the BIG test. This alignment likely enhances the reliability of the results because children are naturally curious and actively explore the world around them (Burdett and Ronfard 2023; Willard et al. 2019). The BIG test, which includes objects that they rarely encounter in their daily lives, stimulates their exploratory behavior, leading to more authentic responses. This, in turn, helps assess their abilities through natural play actions. Validity testing analysis found that there were significant correlations between the BIG test and the UUT in terms of fluency and originality, but weaker correlations with specific dimensions of the PMT, such as fluency and flexibility, and no significant correlation with the overall PMT score. These findings on validity are consistent with those observed in the UBT, where the modest correlation with the UUT supports the BIG test’s effectiveness in measuring creative thinking (Bijvoet-van Den Berg and Hoicka 2014). The BIG test’s non-verbal, interactive play format emphasizes participants’ divergent thinking and hands-on skills in real situations, which aligns more closely with the UUT, which also focuses on applying creative thinking to solve real-world problems (Ritter and Mostert 2017).

In contrast, the lack of significant correlation between the BIG test and the PMT can be attributed to the distinct focuses of the two assessments. The PMT needs participants to construct connections between images and imagination, which focuses more on visual information and associative thinking (Sarsani 2005). This difference in focus explains why the PMT remains less aligned with the hands-on, action-based approach of the BIG test in Chinese preschoolers.

While our study provides preliminary evidence that the BIG test effectively assesses divergent thinking in preschool-aged children in China, several limitations warrant consideration. The relatively small sample size of 47 children may significantly limit the generalizability and external validity of our findings. Smaller sample sizes inherently reduce statistical power, which can impede the detection of true effects and relationships within the data. Specifically, with a limited number of participants, there is an increased risk of Type II errors (failing to detect an effect that is present), leading to potentially misleading conclusions about the effectiveness and reliability of the BIG test. Moreover, the homogeneity of our sample could further restrict the applicability of our findings to broader populations. Our study primarily included children from specific regions or backgrounds, which might not fully represent the diversity of Chinese preschoolers across different cultural, socioeconomic, and geographic contexts. This limitation underscores the necessity for future research to replicate these studies with larger, more diverse samples. By expanding the sample size and ensuring greater demographic diversity, future studies can enhance the robustness of the results and provide stronger evidence for the validity and reliability of the BIG test. Although the BIG test exhibited good internal consistency and moderate test-retest reliability, challenges remain in establishing its effectiveness as an assessment tool for divergent thinking. Existing literature raises concerns about the validity of traditional divergent thinking methods, which may lead to questions about the BIG test’s reliability in measuring creativity may be questionable. Despite positive correlations with the Unusual Uses Task (UUT) and the Pattern Meanings Test (PMT), these correlations alone do not definitively validate the BIG test’s efficacy. To enhance the validity of the BIG test, further research is essential, including comparative studies with established creative measurement tools and longitudinal studies to assess predictive validity. Additionally, more empirical evidence is needed to determine whether cultural adaptations adequately sufficiently address all cultural differences.

While the BIG test has proven to be a reliable and culturally sensitive tool for assessing divergent thinking in Chinese preschoolers, there are several areas where future research could build upon these findings. First, the current implementation of the BIG test requires manual coding and scoring, which is time consuming and subject to human error. Future research should explore the use of automated scoring methods, such as the automatic recognition of actions through machine learning. Second, children’s cognitive factors could contribute to individual differences in divergent thinking ability in preschoolers. For example, a large body of research on adolescents and adults suggests that executive functions, attention, and intelligence are related to an individual’s creative ability (Benedek et al. 2014; Frith et al. 2021; Liang et al. 2024; Nusbaum and Silvia 2011). A new study showed that both domain-general executive functions played a role in the real-time unfolding of original ideas during the divergent thinking task (Bai et al. 2021). Future research should examine the coordinated and interactive development between divergent thinking and other cognitive abilities in preschoolers and toddlers by collecting data from a larger sample size. Additionally, examining the role of environmental factors, such as family dynamics and parental involvement, could provide valuable insights into how these factors shape the development of divergent thinking. Understanding these relationships could guide the development of interventions aimed at nurturing creativity from an early age.

## 5. Conclusions

Preliminary evidence suggests that the BIG test provides a promising and culturally appropriate approach for assessing divergent thinking in Chinese preschoolers. By capturing young children’s creative abilities through non-verbal, action-based interactions, it addresses key limitations of traditional verbal tasks. The current findings support the test’s acceptable reliability and offer initial support for its validity. However, given the modest effect sizes observed, further refinement and validation are needed. Future developments—such as incorporating automated scoring methods—may improve the test’s precision and broaden its applicability in research on early childhood creativity.

## Figures and Tables

**Table 1:** Descriptive statistics for the pre-test scores across three age groups.

Group	Sample Size	Age	PMT_ flu	PMT_ flex	PMT_ ori	UUT_ flu	UUT_ flex	UUT_ ori	BIG_flu	BIG_ori
3–4 y	14	3.43 ± 0.39	2.70 ± 1.40	1.80 ± 1.28	1.05 ± 0.72	2.21 ± 1.16	2.43 ± 1.28	7.43 ± 6.20	35.64 ± 9.95	60.67 ± 20.69
4–5 y	11	4.86 ± 0.23	3.98 ± 3.11	3.61 ± 3.13	2.14 ± 1.93	3.32 ± 1.59	3.27 ± 1.60	10.32 ± 7.15	37.39 ± 13.06	66.36 ± 28.77
5–6.5 y	22	5.75 ± 0.34	3.10 ± 2.12	2.62 ± 1.60	1.84 ± 1.39	3.05 ± 1.53	3.11 ± 1.49	9.20 ± 6.39	45.59 ± 11.43	78.80 ± 22.87
Total	47	4.84 ± 1.05	3.19 ± 2.22	2.61 ± 2.05	4.79 ± 8.22	2.86 ± 1.48	2.95 ± 1.47	8.94 ± 6.47	39.55 ± 12.29	70.33 ± 26.48

Note: PMT, Pattern Meanings Test; UUT, Unusual Uses Task; BIG, Box Interaction Game; flu, fluency; flex, flexibility; ori, originality.

## Data Availability

Data are available on request from Qunlin Chen.

## Appendix A. Sample Scale: Subject 1—Egg Stand

**Table A1:** Scoring table.

	Round Hole	Rectangular Room	Black Baffle	Gap	Stairs	Blocks	Rings	Strings	Edge of the Box	Side of the Box	Iron Wire	Whole Box	Out of the Box
Jump													
Walk		1											
Hit													
Roll													
Flip roll													
Turn													
Revolve													
Drop	2												
Pass through													
Touch							3						
Hold in place													
Place													
Pull													
Drag/stretch													
Squeeze													
Throw against													
Shake													
Whisk													
Curl													
Poke													
Hang													
Dial													
Connect													
Plane													
Flight													4
Write													
Slide													

Action 1: Use the egg stand to “walk” in “the rectangular room”. Action 2: Use the egg stand to “drop” in “the round hole”. Action 3: Use the egg stand to “touch” the “rings”. Action 4: No interaction with the box, just playing with the egg sand.

## Appendix B. Detailed Grading Rule

**Table A2:** Detailed Grading Rule.

Actions	Description
Jump (old action)	During a 2 s interval, the object sequentially contacts (part of) the box two or more times. After these consecutive contacts, the object is elevated to a height greater than necessary for normal walking. The object continues to be held throughout the jumping action.
Walk (old action)	During the interaction, a continuous displacement is initiated upon the object contacting (part of) the box. Throughout the subsequent walking motion, the object is consistently maintained in hand.
Hit (old action)	The object strikes the box one or more times, making rapid and forceful contact with the box in a downward motion from top to bottom.
Roll (old action)	The object is rolled over the surface of the box; the object is either held or let go.
Flip roll (new action)	The object rolls along the surface of the box, performing a flipping motion.
Turn (old action)	The object is turned around.
Revolve (new action)	The object rotates around an axis that is perpendicular to the contact surface. This motion can be likened to rotating an egg rack when placing it into a box.
Drop (old action)	The object is held above the place where it will land and then is let go.
Pass through (new action)	While holding the object, it is guided through (part of) the box without stopping.
Touch (old action)	During the interaction, the object momentarily contacted the box without releasing the grasp. Specifically, the object remained continuously held in hand throughout the brief contact event.
Hold in place (old action)	The object remained in contact with the box for an extended period while being continuously held.
Place (old action)	The object makes contact with the box and is then released, after which it remains stable and independent for a period of time.
Pull (old action)	During the interaction, a (partial) section of the box or object is subjected to a pushing force. Specifically, only a portion of the box or object is pressed or moved in response to this applied force.
Drag/Stretch (new action)	The (partial) movement of the box/object towards the participant.
Squeeze (old action)	The object is squeezed, using thumb and index finger.
Throw against (old action)	The object is thrown against the box.
Shake (old action)	During the interaction, the object is securely held in the hand and is moved swiftly from side to side. This motion does not involve any contact with the box but allows for the accurate determination of the box’s position.
Whisk (new action)	Walk back and forth over the same area on the surface of the box.
Curl (new action)	In this procedure, soft objects such as the handle of a racket, a feather, or a strip are bent and deformed. These manipulations involve applying force to alter the shape and structure of the materials.
Poke (new action)	Use the tip to apply forceful contact to the surface or interior of other objects, such as poking egg holders with strips or rubber toys or poking the side of the box with a paddle.
Hang (new action)	Toys are suspended from a section of the box and remain hanging.
Dial (new action)	Using the hand to make brief contact with a part of the box or an object, applying a short burst of force, causing the object to shift a short distance or a part of the box (such as a strip) to move away from its initial position.
Connect (new action)	Use toys to connect the parts of the box, such as connecting the sides of the box with a beat, connecting the stairs to the edge of the box with a beat.
Plane (new action)	In this procedure, the ground is shaped to resemble a digging motion, creating an inclined or structured surface. A force is then applied to the toy, causing it to move from the bottom upwards.
Flight (new action)	Simulate the toy as a flying object and conduct flight experiments.
Write (new action)	Simulate the toy as a writing instrument and conduct writing experiments.
Slide (new action)	Simulate the toy or box as a slide and conduct sliding experiments.
